# Menopausal Hormone Therapy use and breast cancer risk by receptor subtypes: Results from the New South Wales Cancer Lifestyle and EvaluAtion of Risk (CLEAR) study

**DOI:** 10.1371/journal.pone.0205034

**Published:** 2018-11-07

**Authors:** Usha Salagame, Emily Banks, Dianne L. O’Connell, Sam Egger, Karen Canfell

**Affiliations:** 1 Cancer Research Division, Cancer Council NSW, Woolloomooloo, Sydney, New South Wales, Australia; 2 Sydney School of Public Health, The University of Sydney, Sydney, New South Wales, Australia; 3 National Centre for Epidemiology and Population Health, Australian National University, Canberra, Australia; 4 Sax Institute, Sydney, New South Wales, Australia; 5 School of Medicine and Public Health, Faculty of Health and Medicine, University of Newcastle, Callaghan, New South Wales, Australia; 6 Prince of Wales Clinical School, UNSW, Sydney, Australia; Universita degli Studi di Torino, ITALY

## Abstract

Breast cancer risk is increased with current Menopausal Hormone Therapy (MHT) use, with higher risks reported for ER+ (Estrogen Receptor positive), and ER+/PR+ (Estrogen and Progesterone Receptor positive) breast cancers than those of ER- and ER-/PR- status, respectively. There is limited evidence to suggest MHT use is associated with the specific subtype characterised as ER+/PR+/HER2- (Estrogen and Progesterone Receptor positive and Human Epidermal growth factor Receptor2 negative) status. This study aims to investigate the MHT-breast cancer relationship for breast cancer tumor receptor subtypes defined by ER expression alone, by ER and PR expression only and by joint expression of ER, PR, and HER2. Analyses compared 399 cancer registry-verified breast cancer cases with receptor status information and 324 cancer-free controls. We used multinomial logistic regression to estimate adjusted odds ratios (aORs) and 95% Confidence Intervals (CI) for current and past versus never MHT use, for subgroups defined by tumor receptor expression. Current, but not past, use of MHT was associated with an elevated risk of ER+ breast cancer (aOR = 2.04, 95%CI: 1.28–3.24) and ER+/PR+ breast cancer (aOR = 2.29, 1.41–3.72). Current MHT use was also associated with an elevated risk of the ER+/PR+/HER2- subtype (aOR = 2.30, 1.42–3.73). None of the other subtypes based on ER, ER/PR or ER/PR/HER2 expression were significantly associated with current MHT use in this analysis. Current, but not past, use of MHT increases the risk of breast cancer, with consistently higher risks reported for ER+ and ER+/PR+ subtypes and mounting evidence regarding the specific ER+/PR+/HER2- subtype. Our findings contribute to quantification of the effects of MHT, and support efforts to articulate the receptor-mediated mechanisms by which MHT increases the risk of breast cancer.

## Introduction

It is estimated that 1.67 million new cases of breast cancer were diagnosed in 2012, making breast cancer the second most common cancer globally and the most frequent cancer among women [1]. Breast cancer is the fifth most common cause of cancer-related death globally. Estrogen Receptor (ER), Progesterone Receptor (PR), and Human Epidermal growth factor Receptor2 (HER2) are important molecular biomarkers for breast cancer. ER and PR expression in breast cancer cells determine hormone therapy responsiveness [2]. Differences between breast cancers by ER/PR status have been shown in the aetiology [3,4,5,6,7,8,9,10,11], disease progression and prognosis [12], epidemiology [8,13,14,15] and response to available treatments [2,16,17].

There is a large degree of overlap between the immunohistochemical subtypes defined by ER, PR and HER2 status, and those identified by molecular expression studies, including with Ki67 expression (a marker of tumor cell proliferation) used additionally to distinguish between the luminal subtypes [18]. For practical therapeutic purposes, subtypes based on ER, PR and HER2 status, (i.e. positive or negative denoted as + or—hereafter) and Ki67 expression are considered to be surrogates or convenient approximations of the four intrinsic clinically important subtypes which have been identified through multi-gene microarray profiling [18,19]. The ER+/PR+/HER2- phenotype is a surrogate for so-called luminal A intrinsic subtype (when Ki67 expression <14%) and for the luminal B intrinsic subtype (when Ki67 expression ≥14%; this subtype is often characterised by low PR expression), the ER+/PR+ /HER2+ phenotype is also a surrogate for the luminal B subtype. The ER-/ PR-/HER2+ is a surrogate for the HER2 subtype and the triple negative phenotype (ER-/PR-/ HER2-) is a surrogate for the basal subtype of breast cancers [19]. As per the widely accepted 13th St Gallen International Breast Cancer Conference (2013) Expert Panel recommendations, these are referred to as luminal A-like, luminal B-like, HER2 type and triple negative-basal-like subtypes respectively, to indicate that they are proxies of the molecular subtypes [20]. In Australia, the three tumor markers—ER, PR and HER2 are routinely used in the diagnostic workup of breast cancer because of their utility in prognosis and guiding treatment [21], given some analytical problems with Ki67 measurement and standardisation.

Use of Menopausal Hormone Therapy (MHT) is an important modifiable risk factor for breast cancer [22,23,24,25,26]. Prior studies have identified significantly increased risks with current (vs never) MHT use, with higher risks for ER+ than for ER- breast cancers when ER expression only was considered, [27] and for ER+/PR+ breast cancers but not ER-/PR-breast cancers when ER and PR expression was considered [8,28,29]. In the USA, between 2000 and 2004, for women aged 50 years and over, a decline was demonstrated in breast cancer incidence for ER+, but not ER- cancers [30], in the context of major decreases in MHT use which occurred following the publication of the Women’s Health Initiative (WHI) trial; declines in overall breast cancer incidence were subsequently demonstrated in a number of countries including Australia [31,32]. Similar trends for ER+ breast cancers were documented in other US-based studies [33,34].

ER-/PR+ is a relatively rare phenotype, often with very low levels of PR expression, which is frequently not reproducible between assays [35]. ER+/PR- breast cancers are more common than ER-/PR+; some studies have reported elevated risks associated with current MHT use for this phenotype with point estimates which were intermediate between those for ER+PR+ and ER-PR- [8,28], although heterogeneity in risk by joint ER/PR status was not always established.

For Australian women enrolled in the Melbourne Collaborative Cohort Study, compared to never use, recent MHT use (current or last use in the past year) was associated with a significantly increased risk for ER+ but not ER- breast cancers (when examined by ER status alone), for PR+ but not PR- breast cancers (when examined by PR status alone) and for both ER+/PR+ and ER-/PR-breast cancers, although there was no statistical heterogeneity in the hazard ratios across subtypes defined by ER, PR or ER/PR status (n = 336 breast cancers in a cohort of 13,444 women) [36].

With respect to joint expression of ER, PR and HER2, evidence is limited. An increased breast cancer risk in current versus never-users of MHT for the ER+/PR+/HER2- subtype has been reported in the Nurses’ Health Study and California Teachers’ Cohort Study and from a Norwegian screening program nested case-control study [29,37,38]. The associations between MHT use and subtypes involving HER2 expression have not been examined previously in an Australian setting.

A substantial proportion of menopausal women continue to use MHT, in Australia [39,40,41] and elsewhere [42,43]. Breast cancer risk increases with increasing duration of use of MHT [25]. A total of around 500,000 women in Australia were estimated to use MHT in 2013–14, which includes 13% of Australian women aged 50–69 with ~75% of these women using MHT for ≥ 5 years [39,41,44]. Therefore, use of MHT, and the risks associated with its use, remains an important issue in clinical practice in these settings.

We have previously reported findings from a case-control study {the New South Wales Cancer Lifestyle and EvaluAtion of Risk (NSW CLEAR) study} showing that the risk of breast cancer was doubled with current, but not past use of MHT [45]. Here, we describe further analyses using a subset of cancer registry-verified CLEAR breast cancer cases with hormone-receptor status information available and cancer-free controls recruited over the same period. The aim of this analysis was to investigate the relationship between use of MHT and breast cancer tumor receptor subtypes defined by ER expression alone, by ER and PR expression, and by the joint expression of ER, PR, and HER2. Ki67 data for this subset of women was not always reported and data completeness was low; thus, in the absence of Ki67 data, we mapped the ER+/PR+ phenotype to the best possible approximations of the intrinsic luminal subtypes. The ER+/PR+/HER2- phenotype was here approximated and termed as luminal (HER2-) and the ER+/PR+/HER2+ phenotype as the luminal (HER2+) rather than the luminal A and B subtypes. We hypothesized, based on prior findings, that the odds of breast cancer for current versus never MHT users would be higher for ER+ and ER+/PR+ subtypes and the ER+/PR+/HER2- subtype than for the corresponding receptor-negative breast cancers.

## Methods

### Study design

We used data from the NSW CLEAR Study, an (all-cancer types) case—(cancer-free) control study sponsored by Cancer Council NSW, a not-for-profit cancer control organisation. Between 2006 and 2014, the study recruited NSW residents (from a population of ~7.5 million, and an area of 809,444 sq. km), aged 18 years or over, with a self-reported first diagnosis in the previous 18 months of any type of invasive cancer, or who were recruited as cancer-free controls [46]. The CLEAR study employed an ‘all cancer case-spouse control’ design, whereby cancer-free partners of cases diagnosed with a variety of cancers were recruited as potential controls. This approach provided a pool of same-sex controls for each cancer case, and has been used successfully in previous studies [47,48]. For analyses requiring sex-matching (as for the current analysis), female controls were selected for comparison with female breast cancer cases. Participants were requested to answer the questions ‘thinking of the time just before’ they (for cases) or their partners (for controls) were diagnosed with cancer. Therefore, the date of cancer diagnosis for the cases, or the date of the partner’s cancer diagnosis for the controls, was used as the reference ‘index’ date. Self-reported cancer diagnosis and timing was verified by comparison with cancer registrations in the NSW Cancer Registry (NSWCR) via routine annual record linkage.

Recruitment was conducted via a targeted approach (using a medical or health-related database to identify and generate a list of potential cases) and a non-targeted approach (through widespread promotion at community events, through websites or through face-to-face recruitment at certain oncology clinics). All participants were aged 18 years or over at the time of providing consent; participation in the study was completely voluntary. Written informed consent was obtained from all participants; separate information sheets and consent forms were administered to cases and their partners. The overall consent procedure and the consent forms were reviewed and approved by the St. Vincent’s Hospital Human Research Ethics committee which was the committee responsible for the conduct of this study.

For consenting participants, separate questionnaires were administered to men and women at recruitment, and self-reported information including age, height, weight and weight gain, smoking, physical activity, diet, cancer screening behaviour, medications, medical history, occupation and sun exposure was collected. For women, additional information was collected on reproductive history, history of hysterectomy or oophorectomy, menstrual history and history of use of oral contraceptives and MHT. Consent to participate in the study included consent to link questionnaire data to data from administrative health records, and participants could separately opt to provide a blood sample.

### The analysis dataset for MHT use and breast cancer risk by receptor subtype

The following criteria applied for breast cancer cases included in the analysis: 1) self-report of a first diagnosis of primary invasive breast cancer with no prior cancer diagnosis; 2) enrolled in the study within 18 months of self-reported diagnosis, 3) self-reported diagnosis year of 2008 or before (at the time of the pathology data extraction (January 2013), only cancers diagnosed in 2008 or earlier were able to be linked to the NSWCR for verification of diagnosis due to the timing of cancer registry data becoming available); 4) confirmed by the NSWCR as having breast cancer diagnosed with an ICD code of ‘C50’; 5) have pathology information available for ER/PR status (and possibly HER2 status); and 6) postmenopausal at the time of recruitment. The following criteria applied for controls: 1) self-reported as never diagnosed with cancer; 2) enrolled within 18 months of their partner’s diagnosis; 3) recruited during the same time period as the cases in this study; 4) verified as having no cancer registration in the NSWCR at the time of record linkage; and 5) postmenopausal at the time of recruitment.

Additional methods used to: 1) determine menopausal status of participating women; 2) extract receptor status data and link to registry records and the CLEAR questionnaire dataset; 3) assess completeness and representativeness of receptor status information in the NSWCR pathology data repository are described in Supporting Information S1 File.

### Multinomial logistic regression model to quantify the association between MHT use and breast cancers of specific receptor subtypes

Firstly, a binomial logistic regression model was used to quantify the relationship of current, past versus never use of MHT to breast cancer risk, for all breast cancer subtypes considered together. A multinomial logistic regression analysis was then performed with four levels of the outcome variable (ER+/PR+ (double receptor-positive) cases, ER+/PR- (single receptor-positive) cases, ER-/PR- (double receptor-negative) cases, and cancer-free controls, which was the base-outcome group. ER-/PR+ breast cancers were too few in number for inclusion (6 cases in total) and so were excluded from the current analysis as done in previous studies [29,49]. Odds ratios associated with the current and past use of any type of MHT were estimated for ER+/PR+, ER+/PR- and ER-/PR- breast cancers; never users of MHT constituted the reference group. In addition to these primary comparisons with the ‘never user’ reference group, we also calculated odds ratios for current MHT use for ER+/PR+ and ER+/PR- breast cancers versus ER-/PR-breast cancers (case-case analysis) using the contrast statement (SAS 9.3).

In another multinomial logistic regression analysis, odds ratios for current, past versus never use of any type of MHT were estimated based on the joint expression of ER, PR, and HER2. In the absence of Ki67 data, we mapped the ER+/PR+ phenotype to the best possible approximations of the intrinsic luminal subtypes to interpret our findings—the ER+/PR+/HER2- phenotype was approximated as luminal (HER2-) and the ER+/PR+/HER2+ phenotype as luminal (HER2+) (rather than the luminal A and B subtypes). ER-/PR-/HER2+ and ER-/PR-/HER2- were used as surrogates of the related intrinsic subtypes i.e. HER2 type and basal-like respectively. Cancer-free controls were used as the base outcome group.

For all analyses, the odds ratios were adjusted for age on the index date (18–54 years or ≥ 65 years versus 55–64 years), childbearing history, family history of breast cancer, place of residence (based on ARIA+ (Accessibility and Remoteness Index of Australia), socio-economic disadvantage quintile, BMI on the index date, use of hormonal contraceptives, number of alcoholic drinks consumed per week, time since menopause, and the number of self-reported mammographic screening events in the last ten years. For the cases, we excluded mammographic screening events that were presumed to be associated with the recent diagnosis of breast cancer (i.e. within the 12 months prior to diagnosis) in determining their screening history. All the variables were categorical; variables with greater than 4% missing data (which were BMI and oral contraceptive use) were retained and the missing values treated as a separate category [45].

All analyses were performed using SAS software, version 9.3 (SAS Institute Inc., Cary, NC, USA.).

### Ethics approvals

The design and conduct of the CLEAR study including the questionnaire, recruitment and data collection were ethically approved by St. Vincent’s Hospital Human Research Ethics Committee (HREC 07/SVH/106, 20.12.07). The current case-control analysis of MHT use and breast cancer risk by receptor subtype was peer-reviewed by the CLEAR Expert Advisory Committee (March 2012) and ethically approved by the NSW Population and Health Services Research Ethics Committee (HREC/11/CIPHS/53) and the University of Sydney Human Research Ethics Committee (2013/538). Data extraction from the pathology reports was executed through a MoU (26-10-2012) between Cancer Council NSW and Cancer Institute NSW (the data custodians). All procedures performed in studies involving human participants were in accordance with the ethical standards of the institutional and/or national research committee and with the 1964 Helsinki declaration and its later amendments or comparable ethical standards. Informed consent was obtained from all individual participants included in the study.

## Results

### Completeness and representativeness of receptor status information in the collection of pathology reports held at the NSW cancer registry

**Fig 1:**
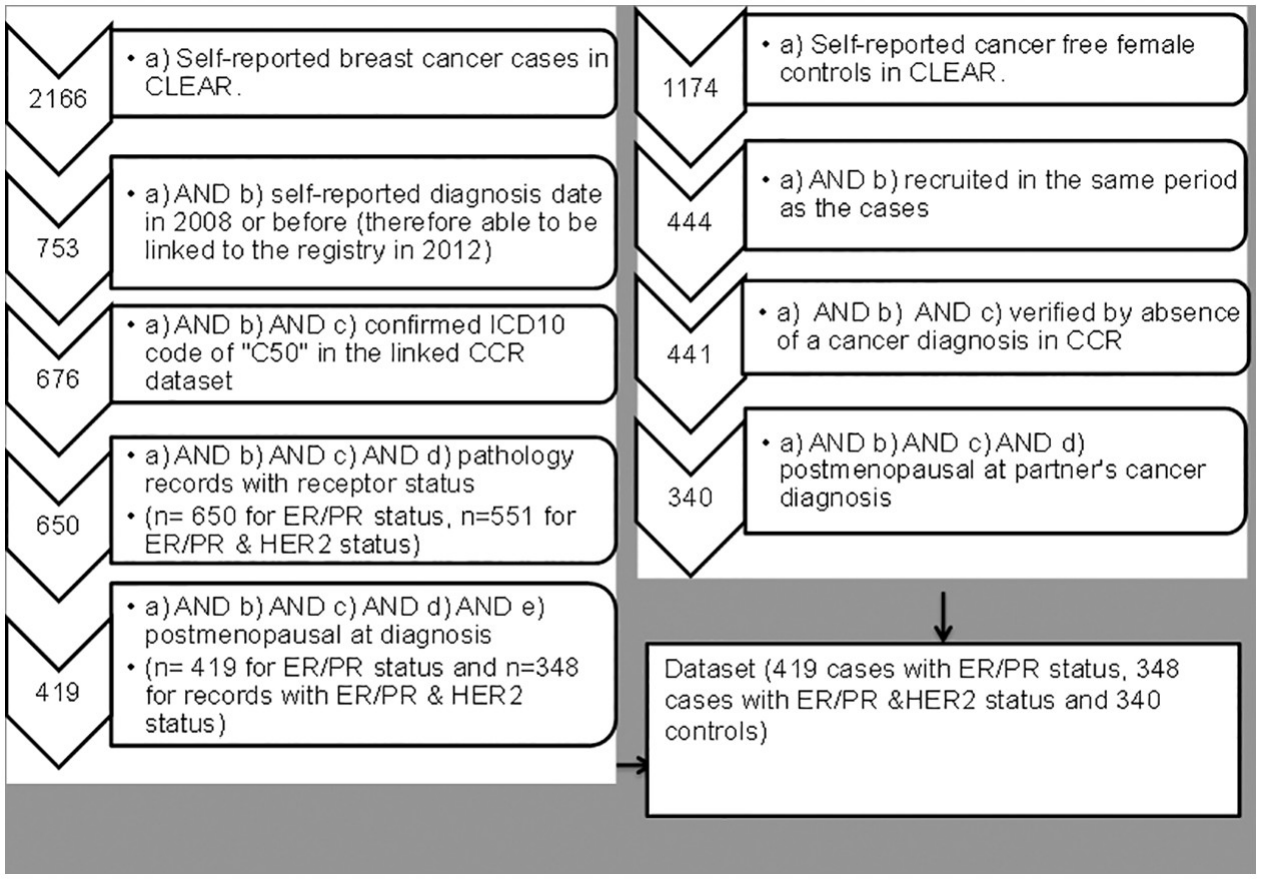
Available and eligible cases and controls for the analysis of MHT and breast cancer risk by receptor subtype.

Pathology data with ER and PR receptor status were available for 650 of the 676 self-identified and registry verified CLEAR breast cancer cases, with 419 of these categorised as postmenopausal (Fig 1). ER, PR, and HER2 status was available for 551 women (with 348 categorised as postmenopausal). A total of 340 controls categorised as postmenopausal were verified as being cancer-free via confirmation of an absence of a cancer diagnosis record in the cancer registry.

Details of the receptor status data extraction from the pathology reports in the NSW Cancer registry are provided in Supporting Information S1 File. Overall, the completeness of receptor status information for the registry-verified CLEAR cases was very high; 96% for ER and PR status and 85% for HER2 status, including 3% of cases with ‘equivocal’ HER2 status (Table A in S1 File). Information on the method of HER2 detection was not extracted; hence it is not known if cases with equivocal results for HER2 were tested by Immunohistochemistry or Fluorescence In-Situ Hybridisation.

Of the cases with known ER and PR status, 83.5% of all CLEAR cases (and 83% of the postmenopausal cases) were positive for at least one receptor and 17% of all cases and all postmenopausal cases were both ER and PR negative (Table B in S1 File). For the proportional distribution by joint ER, PR, and HER2 overexpression, 72% of all cases (and 70% of postmenopausal cases) were of the ER+/PR+/HER2-subtype, 10% of all cases were of the ER+/PR+/HER2+ subtype, 6% were ER-/PR-/HER2+ and 12% were negative for all three receptors.

### MHT use and invasive breast cancer risk for the hormone receptor subtypes and the surrogate clinical subtypes

**Fig 2:**
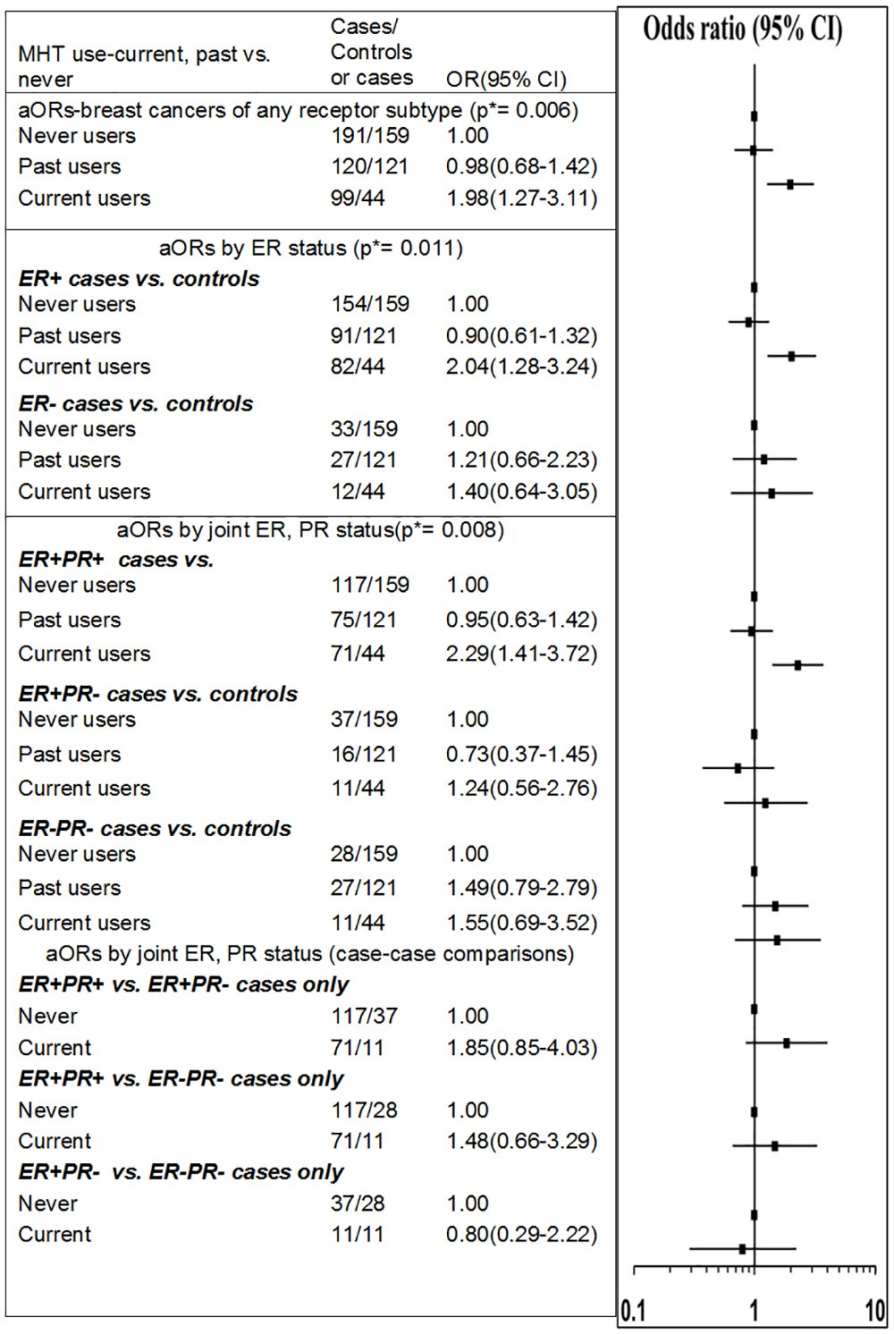
MHT use and invasive breast cancer risk by breast cancer hormone receptor (ER/PR) status. Legend: p* = p-value is for the test of global null hypothesis that the OR estimates are equal to one (i.e their reference group OR). Breast cancers with ER-/PR+ status (n = 6) were excluded from the analyses. All odds ratios were adjusted for age at index date, BMI, parity cross-classified by age at first birth, time since menopause, family history of breast cancer, place of residence, socioeconomic disadvantage quintile, oral contraceptive use, history of breast screening and alcohol consumption.

After excluding cases and controls with missing values for one or more of the explanatory variables, a total of 399 cases and 324 controls were included in the final multivariable analysis for breast cancer by ER/PR status and 332 cases and 324 controls were included in the analysis for surrogate clinical subtypes. The risk of any type of breast cancer was found to be increased significantly for current, but not past, users of any type of MHT. In a binomial model, compared to never users, current users had a significantly higher odds of breast cancer (aOR = 1.98, 95%CI: 1.27–3.11), whereas, for past users, the odds were not significantly different from that for never users (aOR = 0.98, 95%CI: 0.68–1.42) (Fig 2). These estimates are similar to those reported previously for current and past users in the larger CLEAR dataset [45].

In a multinomial model, with breast cancers defined by ER status alone, current use (versus never use) was significantly associated with the ER+ (aOR = 2.04, 95%CI: 1.28–3.24), but not the ER- breast cancers (aOR = 1.40, 95%CI: 0.64–3.05). A significant difference between the odds ratios for the ER+ and ER-breast cancers was not identified in the case-case comparison (p = 0.325). In another multinomial model, with breast cancer subtypes defined by joint ER and PR status, current use was significantly associated with ER+/PR+ status only (aOR = 2.29, 95%CI: 1.41–3.72). No significant differences in the odds ratios between ER+/PR+, ER+/PR- and ER-/ER- subtypes were found through the case-case comparisons shown in Fig 2. However, this may be due to limited statistical power to detect significant differences in these data. Past use was not found to be significantly associated with increased odds of breast cancer for any the subtypes defined by ER/PR status (Fig 2).

**Fig 3:**
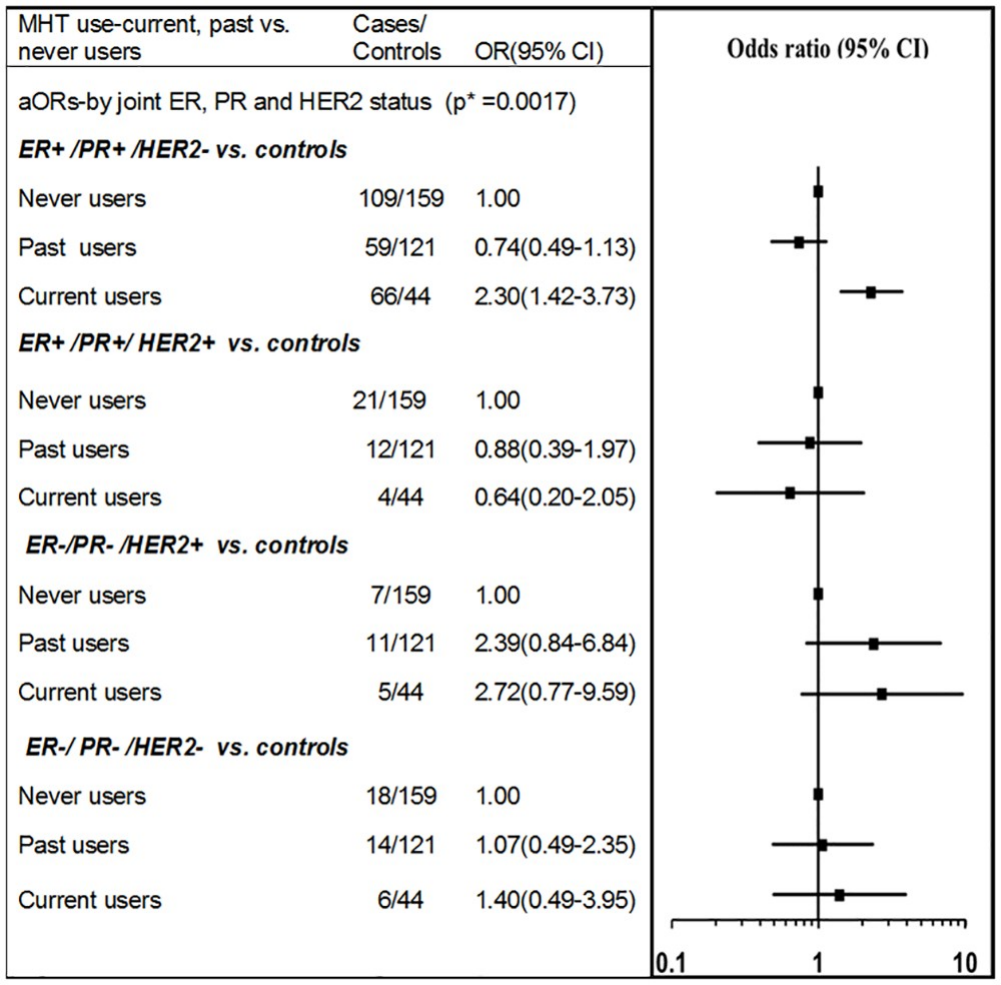
MHT use and invasive breast cancer risk for subtypes based on ER, PR, and HER2 status. P* = p-value is for the test of global null hypothesis that the OR estimates are equal to one (i.e their reference group OR). All odds ratios were adjusted for age at index date, BMI, parity cross-classified by age at first birth, time since menopause, family history of breast cancer, place of residence, socioeconomic disadvantage quintile, oral contraceptive use, breast screening history and alcohol consumption.

With respect to an association with HER2 status alone, current use of any type of MHT was found to be associated with a significant elevation in the risk of HER2- breast cancer (aOR = 2.20, 95%CI: 1.36–3.55), but not HER2+breast cancer, although it should be noted that in the current analysis the odds ratios by HER2 expression were not significantly different (p(het) = 0.183). Quantitative estimates for an association between MHT use and the subtypes based on joint expression of ER, PR, and HER2 were tested in a multivariable model (Fig 3). Current use of MHT was found to be associated with the ER+/PR+/HER2- phenotype (aOR = 2.30, 95%CI: 1.42–3.73) (Fig 3). None of the other types of breast cancer were found to be significantly associated with current MHT use. For current users of MHT, a significant difference in the odds of breast cancer was detected for the ER+/PR+/HER2- versus ER+/PR+/HER2+ subtypes [case-case analysis: ER+/PR+/HER2+ versus ER+/PR+/HER2- (aOR = 0.28, 95%CI: 0.09–0.88, p = 0.029)], although the odds ratios were not significantly different across any of the other subtypes: ER+/PR+/HER2- vs. ER-/PR-/HER2+(p(het) = 0.793); ER+/PR+/HER2- vs. ER-/PR-/HER2-+(p(het) = 0.342).

## Discussion and conclusions

In the current analysis we have found that current users of MHT have approximately double the odds of developing ER+ breast cancer compared to women who had never used MHT. There was also a 2.3 fold increase in the odds of developing ER+PR+ and ER+/PR+/HER2- subtypes of breast cancer for current versus never users of MHT. None of the other breast cancer subtypes were found to have a significant association with current MHT use in this analysis.

Our findings, using new data and a novel study design, provide independent replication and verification of previous findings of increased risk of breast cancer generally, and ER+ breast cancers specifically, for current versus never users of MHT [27]. Our results are consistent with prior work in the USA, in which ecological studies have reported declines in the population level incidence of ER+ cancers concurrent with the widespread decline in MHT use in the population during the period following the publication of the first results from the WHI trial [29,30,33,34]. An increased risk of the ER+/PR+ double-receptor-positive subtype for current users versus never users of MHT, as we found, was previously reported in the Nurses’ Health Study [28], the California Cohort Study [29] and the Melbourne Collaborative Cohort Study [36].

This is one of the few studies internationally and the first Australian study to examine MHT-associated breast cancer risk in relation to approximations of surrogate clinical subtypes based on the joint expression of ER, PR, and HER2, and to demonstrate an association between MHT use and the ER+/PR+/HER2- (our approximated surrogate for luminal (HER2-) breast cancer subtype. Tamimi et al have previously reported ORs of 1.40(1.10–1.70) and 1.50(1.20–2.00) for current estrogen-only and estrogen-progestagen combination therapy use for the luminal A-like subtype in the Nurses’ Health Study [37]. Ellingjord-Dale et al have reported an OR of 2.92(2.36–3.62) for the ER+/PR+/HER2- phenotype for current users of estrogen-progestagen combination therapy in a Norwegian study [38] and Saxena et al have reported ORs ranging from 1.77(1.41–2.21) to 2.07(1.38, 3.09) depending on the duration of current use, for the ER+/PR+/HER2- phenotype in current users of combination therapy, in the California Teachers’ Cohort Study [29]. Our finding for the ER+/PR+/HER2- subtype (aOR = 2.30, 95%CI: 1.42–3.73) is consistent with these prior findings.

In the current study we demonstrated that the collection of pathology records held by the NSWCR was 96% complete for ER and PR status and 85% complete for HER2 status. With respect to the distribution of incident breast cancer by ER and PR status, our findings from the CLEAR study are consistent with those from other contemporary reports [13,50] despite any underlying differences in ethnicity and other demographic and risk factor variables. For example, in the current dataset, our estimate of either ER and/or PR positivity in 83% of cases is consistent with estimates recently reported from the 2010 US Cancer registry SEER (Surveillance, Epidemiology, and End Results program) data in which 83% of cases were positive for ER and/or PR, and 17% were negative for both these markers [13]. The proportions of the four subtypes—ER+/PR+/HER2-, ER+/PR+/HER2+, ER-/PR-/HER2+, ER-/PR-/HER2- in the CLEAR dataset were 72%, 10%, 6% and 12% respectively. These proportions are generally comparable with other reports [51], including recent estimates based on SEER data from 17 USA registries wherein 73%, 10%, 5% and 12% were reported for these subtypes respectively [13]. However, the proportion of receptor-positive breast cancers in the current analysis dataset (83%) was somewhat higher than those previously reported for Australian women (71–77%), whereas, the proportion of double receptor-negative cancers (ER-PR-) in the analysis dataset (17%) was somewhat lower (23–29%) than those analyses [51,52,53], although confidence intervals were not available in the previous reports. The apparent difference could be due to sampling error, due to changing technologies and more sensitive methods for detection of ER and PR, other unknown reasons, or it could reflect a real difference in the mix of breast cancer subtypes in different population subgroups. Further studies with more recent data and larger sample sizes may be needed to confirm this observation. The representativeness of our study sample with respect to cancer stage at diagnosis was ascertained by comparing the distribution of stage at diagnosis data in the study sample with that for the population cancer registry data. We found that the proportions of breast cancers with local and regional disease in the CLEAR dataset were comparable to those reported for the NSW population, although metastatic disease was slightly underrepresented (Table C in S1 File). Another strength of this study is that the findings are based on a contemporary sample of women for whom extensive information on hormonal exposure, reproductive history and demographic characteristics were available. This detailed information made it possible to design a robust multivariable model adjusting for the well-established breast cancer risk factors.

The most significant limitation of this study was sample size and unavailability of Ki67 data. The Royal College of Pathologists of Australia protocol for synoptic reporting of breast cancer does not mandate the reporting of Ki 67 expression [21]. We found that the reporting of Ki67 in the collection of pathology reports was not uniform and infrequently reported. Because of this limitation we were unable to make a distinction between the luminal A-like (more endocrine sensitive, indolent, better prognosis) and luminal B-like (less endocrine sensitive, more aggressive, worse prognosis) subtypes. We used the available ER, PR and HER2 information to categorize breast cancers into subtypes that best approximated the subtypes of luminal, triple-negative, and HER2-overexpressing tumors; a similar approach was used in other previous studies on MHT use and breast cancer risk [29,38].

Although we were able to detect a significant association with current MHT use for the ER+/PR+/HER2- subtype, with cancer-free controls as the reference group, the numbers of cases with pathology data in the HER2 type and basal-like surrogate subtypes who were also current users of MHT were small. Although we found significant differences in the odds of breast cancer between our approximated surrogates for the luminal (HER2-) and luminal (HER2+) subtypes, the odds ratios for the other groups were not significantly different, possibly due to limited statistical power. Analysis by type of MHT preparation was also limited by sample size; in particular, insufficient numbers of receptor-negative cases in users of combination therapy precluded analysis stratified by both MHT type and receptor status.

It should be noted, however, that our dataset included cases diagnosed until the end of 2008 only. With ongoing improvements in detection technologies and the release of guidelines for pathological testing and structured reporting of breast cancers (most recently updated by the Royal College of Pathologists of Australasia in 2012) [21], it is likely that pathology data for breast cancers diagnosed in more recent years is even more complete and of a higher quality. Future linkages with more recent registry data could potentially allow a more detailed stratified analysis.

We demonstrated an association between current MHT use and breast cancers with an absence of HER2 overexpression. The evidence in relation to MHT use and HER2 overexpression is not definitive. MHT use is found to be associated with the luminal A-like subtype which is HER2- [29,37], yet there is some indirect evidence for an association between MHT use and HER2 overexpressing subtypes [54]. HER2 overexpression is an important factor in guiding the course of treatment for breast cancer. Trastuzumab (an antibody preparation against HER2) is an effective targeted treatment specific for HER2 overexpressing breast cancers. In Australia, breast cancers with HER2 gene copy number > 6.0 (as determined by FISH) are considered as HER2 overexpressing and are eligible (since 1^st^ October 2006) for subsidised Trastuzumab therapy as part of the ‘Herceptin program’ administered by the Department of Human Services. In this context, the lack of association between MHT use and HER2 overexpressing breast cancers demonstrated in this study is important information.

The International Agency for Research on Cancer, in their 2012 synthesis of the available evidence on the carcinogenicity of oestrogens, concluded that receptor-mediated responses to hormones are a plausible and probably necessary mechanism for oestrogen and oestrogen-progestagen related carcinogenesis [55,56]. The significantly increased risks associated with the ER+/PR+ breast cancer subtype found in this study, and by others, reinforces the mechanistic plausibility of an association between MHT use and the increased risk of development of hormone receptor-positive breast cancers; wherein the hormonal preparations potentially exert their neoplastic effects through intracellular signalling mediated by the receptors. Our findings suggest that breast cancers related to use of MHT are more likely to express ER and PR receptors. Given the continuing use of MHT, the findings from this analysis are important to inform clinicians of the increased risk for ER+ breast cancers associated with current MHT use by Australian women. They provide additional support to the current recommendations and help to reinforce the current messaging around limiting the use of MHT for the shortest time possible, for the alleviation of moderate to severe menopausal symptoms in women who are informed of the risks and benefits.

## Supporting information

S1 File Supplementary methods and result tables. (DOCX)

## References

[1] Ferlay J, Soerjomataram I, Dikshit R, Eser S, Mathers C, Rebelo M, et al Cancer incidence and mortality worldwide: Sources, methods and major patterns in GLOBOCAN 2012. Int J Cancer 2015;136: E359–E386. 10.1002/ijc.29210 25220842

[2] Rastelli F, Crispino S. Factors predictive of response to hormone therapy in breast cancer. Tumori 2008; 94: 370–383. 18705406 10.1177/030089160809400314

[3] Collaborative Group on Hormonal Factors in Breast Cancer. Menarche, menopause, and breast cancer risk: individual participant meta-analysis, including 118 964 women with breast cancer from 117 epidemiological studies. Lancet Oncol. 2012;13: 1141–1151. 10.1016/S1470-2045(12)70425-4 23084519 PMC3488186

[4] Anderson KN, Schwab RB, Martinez ME. Reproductive risk factors and breast cancer subtypes: a review of the literature. Breast Cancer Res Treat. 2014;144: 1–10. 10.1007/s10549-014-2852-7 24477977 PMC4026199

[5] Colditz GA, Rosner BA, Chen WY, Holmes MD, Hankinson SE. Risk Factors for Breast Cancer according to Estrogen and Progesterone Receptor Status. J Natl Cancer Inst. 2004; 96: 218–228. 14759989 10.1093/jnci/djh025

[6] Ma H, Bernstein L, Pike MC, Ursin G. Reproductive factors and breast cancer risk according to joint estrogen and progesterone receptor status: a meta-analysis of epidemiological studies. Breast Cancer Res. 2006; 8: R43 10.1186/bcr1525 16859501 PMC1779465

[7] Munsell MF, Sprague BL, Berry DA, Chisholm G, Trentham-Dietz A. Body mass index and breast cancer risk according to postmenopausal estrogen-progestin use and hormone receptor status. Epidemiol Rev. 2014; 36: 114–136. 10.1093/epirev/mxt010 24375928 PMC3873844

[8] Setiawan VW, Monroe KR, Wilkens LR, Kolonel LN, Pike MC, Henderson BE. Breast cancer risk factors defined by estrogen and progesterone receptor status: the multiethnic cohort study. Am J Epidemiol. 2009;169: 1251–1259. 10.1093/aje/kwp036 19318616 PMC2727208

[9] Suzuki R, Orsini N, Mignone L, Saji S, Wolk A. Alcohol intake and risk of breast cancer defined by estrogen and progesterone receptor status—a meta-analysis of epidemiological studies. Int J Cancer 2008; 122: 1832–1841. 10.1002/ijc.23184 18067133

[10] Suzuki R, Orsini N, Saji S, Key TJ, Wolk A. Body weight and incidence of breast cancer defined by estrogen and progesterone receptor status—a meta-analysis. Int J Cancer. 2009;124: 698–712. 10.1002/ijc.23943 18988226

[11] Yang XR, Chang-Claude J, Goode EL, Couch FJ, Nevanlinna H, Milne RL, et al Associations of breast cancer risk factors with tumor subtypes: a pooled analysis from the Breast Cancer Association Consortium studies. J Natl Cancer Inst. 2011;103: 250–263. 10.1093/jnci/djq526 21191117 PMC3107570

[12] Rakha EA, El-Sayed ME, Green AR, Paish EC, Powe DG, Gee J, et al Biologic and clinical characteristics of breast cancer with single hormone receptor–positive phenotype. J Clin Oncol. 2007; 25: 4772–4778. 10.1200/JCO.2007.12.2747 17876012

[13] Howlader N, Altekruse SF, Li CI, Chen VW, Clarke CA, Ries LA, et al US Incidence of Breast Cancer Subtypes Defined by Joint Hormone Receptor and HER2 Status. J Natl Cancer Inst. 2014;106: dju055. 10.1093/jnci/dju055 PMC4580552 24777111

[14] Boyle P. Triple-negative breast cancer: epidemiological considerations and recommendations. Ann Oncol. 2012; 23: vi7–vi12. 10.1093/annonc/mds187 23012306

[15] Kwan ML, Kushi LH, Weltzien E, Maring B, Kutner SE, Fulton RS, et al Epidemiology of breast cancer subtypes in two prospective cohort studies of breast survivors. Breast Cancer Res. 2009; 11: R31 10.1186/bcr2261 19463150 PMC2716499

[16] Liedtke C, Mazouni C, Hess KR, André F, Tordai A, Mejia JA, et al Response to neoadjuvant therapy and long-term survival in patients with triple-negative breast cancer. J Clin Oncol. 2008; 26: 1275–1281. 10.1200/JCO.2007.14.4147 18250347

[17] Dignam JJ, Dukic VM, Anderson SJ, Mamounas EP, Wickerham DL, Wolmark N. Hazard of recurrence and adjuvant treatment effects over time in lymph node-negative breast cancer. Breast Cancer Res Treat. 2009; 116: 595–602. 10.1007/s10549-008-0200-5 18830816 PMC2711214

[18] Perou CM, Sorlie T, Eisen MB, van-de-Rijn M, Jeffrey SS, Rees CA, et al Molecular portraits of human breast tumours. Nature. 2000; 406: 747–752. 10.1038/35021093 10963602

[19] Goldhirsch A, Wood WC, Coates AS, Gelber RD, Thürlimann B, Senn HJ, et al Strategies for subtypes-dealing with the diversity of breast cancer: highlights of the St Gallen International Expert Consensus on the Primary Therapy of Early Breast Cancer 2011. Ann Oncol. 2011; 22: 1736–1747. 10.1093/annonc/mdr304 21709140 PMC3144634

[20] Goldhirsch A, Winer EP, Coates AS, Gelber RD, Piccart-Gebhart M, Thurlmann B, et al Personalizing the treatment of women with early breast cancer: highlights of the St Gallen International Expert Consensus on the Primary Therapy of Early Breast Cancer 2013. Ann Oncol. 2013; 9: 2206–23 10.1093/annonc/mdt303 PMC3755334 23917950

[21] The Royal College of Pathologists of Australasia. Invasive breast cancer structured reporting protocol (2nd edition, 2012). https://www.rcpa.edu.au/getattachment/7b70b3e5-5dca-403f-893e-638815f487b1/Protocol-invasive-breast-cancer.aspx (accessed May 2018).

[22] Drug Safety Update.2007; 1(2). http://webarchive.nationalarchives.gov.uk/20080527191907/http://mhra.gov.uk/Publications/Safetyguidance/DrugSafetyUpdate/CON2032234. (accessed May 2018)

[23] UK Public Assessment Report Hormone-replacement therapy: safety update.2007. http://webarchive.nationalarchives.gov.uk/20080527191907/http://mhra.gov.uk/home/groups/pl-p/documents/websiteresources/con2032228.pdf. (accessed May 2018)

[24] Collaborative Group on Hormonal Factors in Breast Cancer. Breast cancer and hormone replacement therapy: collaborative reanalysis of data from 51 epidemiological studies of 52,705 women with breast cancer and 108,411 women without breast cancer. Lancet. 1997; 350: 1047–1059. 10213546

[25] Beral V and Million Women Study Collaborators. Breast cancer and hormone-replacement therapy in the Million Women Study. Lancet. 2003; 362: 419–427. 12927427 10.1016/s0140-6736(03)14065-2

[26] Salagame U, Canfell K, Banks E. An epidemiological overview of the relationship between hormone replacement therapy and breast cancer. Expert Rev Endocrinol Metab. 2011; 6: 397–409. 10.1586/eem.11.31 30754116

[27] Beral V, Reeves G, Bull D, Green J. for the Million Women Study Collaborators. Breast Cancer Risk in Relation to the Interval Between Menopause and Starting Hormone Therapy. J Natl Cancer Inst. 2011; 103: 1–10. 10.1093/jnci/djq527 PMC3039726 21278356

[28] Chen WY, Hankinson SE, Schnitt SJ, Rosner BA, Holmes MD, Colditz GA. Association of hormone replacement therapy to estrogen and progesterone receptor status in invasive breast carcinoma. Cancer. 2004; 101: 1490–1500. 10.1002/cncr.20499 15378477

[29] Saxena T, Lee E, Henderson KD, Clarke CA, West D, Marshall SF, et al Menopausal hormone therapy and subsequent risk of specific invasive breast cancer subtypes in the California Teachers Study. Cancer Epidemiol Biomarkers Prev. 2010; 19: 2366–2378 10.1158/1055-9965.EPI-10-0162 20699377 PMC2936672

[30] Ravdin PM, Cronin KA, Howlader N, Berg CD, Chlebowski RT, Feuer EJ, et al The Decrease in Breast-Cancer Incidence in 2003 in the United States. N Engl J Med. 2007; 356: 1670–1674. 10.1056/NEJMsr070105 17442911

[31] Canfell K, Banks E, Clements M, Kang Y, Moa A, Armstrong B, et al Sustained lower rates of HRT prescribing and breast cancer incidence in Australia since 2003. Breast Cancer Res Treat. 2009; 117: 671–673. 10.1007/s10549-009-0331-3 19219631

[32] Kumle M. Declining breast cancer incidence and decreased HRT use. Lancet. 2008; 372: 608–610. 10.1016/S0140-6736(08)61255-6 18722851

[33] Glass AG, Lacey JV, Carreon JD, Hoover RN. Breast Cancer Incidence, 1980–2006: Combined Roles of Menopausal Hormone Therapy, Screening Mammography, and Estrogen Receptor Status. J Natl Cancer Inst. 2007; 99: 1152–1161. 10.1093/jnci/djm059 17652280

[34] Kerlikowske K, Miglioretti DL, Buist DSM, Walker R, Carney PA. Declines in invasive breast cancer and use of postmenopausal hormone therapy in a screening mammography population. J Natl Cancer Inst. 2007; 99: 1335–1339. 10.1093/jnci/djm111 17698950

[35] Hefti MM, Hu Rong, Knoblauch NW, Collins LC, Haibe-Kains B, Tamimi RM, et al Estrogen receptor negative/progesterone receptor positive breast cancer is not a reproducible subtype. Breast Cancer Res. 2013; 15: R68 10.1186/bcr3462 23971947 PMC3978610

[36] Gertig DM, Fletcher AS, English DR, MacInnis RJ, Hopper JL, Giles GG. Hormone therapy and breast cancer: what factors modify the association? Menopause. 2006; 13: 178–184. 10.1097/01.gme.0000177317.85887.65 16645531

[37] Tamimi RM, Colditz GA, Hazra A, Baer HJ, Hankinson SE, Rosner B, et al Traditional Breast Cancer Risk Factors in Relation to Molecular Subtypes of Breast Cancer. Breast cancer Res Treat. 2012; 131: 159–167. 10.1007/s10549-011-1702-0 21830014 PMC3237947

[38] Ellingjord-Dale M, Vos L, Tretli S, Hofvind S, dos-Santos-Silva I, Ursin G. Parity, hormones and breast cancer subtypes—results from a large nested case-control study in a national screening program. Breast Cancer Res. 2017; 19: 10 10.1186/s13058-016-0798-x 28114999 PMC5259848

[39] Gartoulla P, Davis SR, Worsley R, Bell RJ. Use of complementary and alternative medicines for menopausal symptoms in Australian women aged 40–65 years. Med J Aust. 2015; 203: 146 26224187 10.5694/mja14.01723

[40] MacLennan AH, Gill TK, Broadbent JL, Taylor AW. Continuing decline in hormone therapy use: population trends over 17 years. Climacteric. 2009;12: 122–130. 10.1080/13697130802666251 19259854

[41] Peng W, Adams J, Hickman L, Sibbritt DW. Complementary/alternative and conventional medicine use amongst menopausal women: Results from the Australian Longitudinal Study on Women’s Health. Maturitas. 2014;79: 340–342. 10.1016/j.maturitas.2014.08.002 25190368

[42] Sprague BL, Trentham-Dietz A, Cronin KA. A sustained decline in postmenopausal hormone use: results from the National Health and Nutrition Examination Survey, 1999–2010. Obstet Gynecol. 2012;120: 595–603. 10.1097/AOG.0b013e318265df42 22914469 PMC3607288

[43] Steinkellner AR, Denison SE, Eldridge SL, Lenzi LL, Chen W, Bowlin SJ. A decade of Postmenopausal Hormone Therapy prescribing in the United States: Long-term effects of the Women’s Health Initiative. Menopause. 2012;19: 616–621. 10.1097/gme.0b013e31824bb039 22648302

[44] Velentzis LS, Banks E, Sitas F, Salagame U, Tan EH, Canfell K. Use of Menopausal Hormone Therapy and Bioidentical Hormone Therapy in Australian Women 50 to 69 Years of Age: Results from a National, Cross-Sectional Study. PLoS One. 2016; 11: e0146494 10.1371/journal.pone.0146494 27008039 PMC4805183

[45] Salagame U, Banks E, Sitas F, Canfell K. Menopausal hormone therapy use and breast cancer risk in Australia: Findings from the New South Wales Cancer, Lifestyle and Evaluation of Risk study. International Journal of Cancer. 2016; 138: 1905–1914. 10.1002/ijc.29942 26599391

[46] Sitas F, Yap S, Egger S, Christian K, Hodgkinson V, Barton M, et al The Cancer, Lifestyle and Evaluation of Risk Study (CLEAR): Rationale and design of an unmatched "case-spouse control" study of over 10,000 participants in New South Wales, Australia. Cancer Epidemiol. 2015; 39:414–423. 10.1016/j.canep.2015.03.006 25892705

[47] Jiang J, Liu B, Nasca PC, Han W, Zou X, Zeng X, et al Comparative study of control selection in a national population -based case-control study: Estimating risk of smoking on cancer deaths in Chinese men. Int J Med Sc. 2009; 6: 329–337. 19918375 10.7150/ijms.6.329 PMC2777271

[48] Liu BQ, Peto R, Chen ZM, Boreham J, Wu YP, Li JY, et al Emerging tobacco hazards in China: 1. Retrospective proportional mortality study of one million deaths. BMJ. 1998; 317: 1411–1422. 9822393 10.1136/bmj.317.7170.1411 PMC28719

[49] Li CI, Malone KE, Porter PL, Weiss NS, Tang MC, Cushing-Haugen KL, et al Relationship between long durations and different regimens of hormone therapy and risk of breast cancer. JAMA. 2003; 289: 3254–3263. 10.1001/jama.289.24.3254 12824206

[50] Kurebayashi J, Miyoshi Y, Ishikawa T, Saji S, Sugie T, Suzuki T, et al Clinicopathological characteristics of breast cancer and trends in the management of breast cancer patients in Japan: Based on the Breast Cancer Registry of the Japanese Breast Cancer Society between 2004 and 2011. Breast Cancer. 2015; 22: 235–244. 10.1007/s12282-015-0599-6 25758809

[51] Francis GD, Dimech M, Giles L, Hopkins A. Frequency and reliability of oestrogen receptor, progesterone receptor and HER2 in breast carcinoma determined by immunohistochemistry in Australasia: results of the RCPA Quality Assurance Program. J Clin Pathol. 2007; 60: 1277–1283. 10.1136/jcp.2006.044701 17259294 PMC2095464

[52] Hähnel R, Spilsbury K. Oestrogen receptors revisited: long-term follow up of over five thousand breast cancer patients. ANZ J Surg. 2004; 74: 957–960. 10.1111/j.1445-1433.2004.03215.x 15550082

[53] McCredie MR, Dite GS, Southey MC, Venter DJ, Giles GG, Hopper JL. Risk factors for breast cancer in young women by oestrogen receptor and progesterone receptor status. Br J Cancer. 2003; 89: 1661–1663. 10.1038/sj.bjc.6601293 14583766 PMC2394423

[54] Cerne JZ, Stegel V, Gersak K, Novakovic S. KRAS rs61764370 is associated with HER2-overexpressed and poorly-differentiated breast cancer in hormone replacement therapy users: a case control study. BMC Cancer. 2012; 12:105 10.1186/1471-2407-12-105 22436609 PMC3342891

[55] IARC working group on the evaluation of carcinogenic risks to humans.Combined Estrogen-Progestogen Contraceptives and Combined Estrogen-Progestogen Menopausal Therapy. Vol 91 IARC Monographs on the Evaluation of Carcinogenic Risks to Humans. 2007; 91: 326–329. PMC4781221 18756632

[56] IARC working group on the evaluation of carcinogenic risks to humans. Pharmaceuticals- A review of human carcinogens. IARC monographs on the evaluation of carcinogenic risks to humans. 2012; 100 A: 219–282.

