# Supplementary material for: Menopausal Hormone Therapy use and breast cancer risk by receptor subtypes: Results from the New South Wales Cancer Lifestyle and EvaluAtion of Risk (CLEAR) study
Source: PLoS One. 2018 Nov 7;13(11):e0205034. doi: 10.1371/journal.pone.0205034 (PMC6221262; doi:10.1371/journal.pone.0205034)
Supplement: S1 File — (DOCX) [file pone.0205034.s001.docx]

Supplementary File: Menopausal Hormone Therapy use and breast cancer risk by receptor subtypes: Results from the New South Wales Cancer Lifestyle and EvaluAtion of Risk study

*Usha G Salagame, Emily Banks, Dianne L O’Connell, Sam Egger , and Karen Canfell.*

Supplementary Methods and Result Tables

1. **Determination of menopausal status**

Self-reported breast cancer cases with a confirmed diagnosis of breast cancer in the NSW Cancer registry and with pathology information on ER, PR and HER2 status available, and self-identified cancer-free controls verified as having no prior registration in the NSWCR at the time of record linkage were selected. These women were classified as being pre-, peri- or postmenopausal, based on whether they reported having regular periods, irregular periods or that their periods had stopped by the index date. For those women who were classified as peri- or post-menopausal, time since menopause was estimated as their age at recruitment less 50 years. For those women for whom natural menopause might have been masked by MHT or hormonal contraceptive use or hysterectomy, we applied the following age criteria - women ≥53 years of age were classified as postmenopausal (90% of the study sample aged ≥ 53 years who had not had a hysterectomy or were not using MHT were postmenopausal) and those between 50 and 52 years of age were assigned to unknown menopausal status, following the approach used previously for the Million Women Study.^1^ Women reporting a prior oophorectomy were classified as postmenopausal if they reported that their periods had stopped and time since menopause was estimated as time since oophorectomy, or else classified by applying the age criteria if they reported having periods on the index date (these were most likely women who might have had a partial oophorectomy). In order to avoid confounding by factors associated with menopausal status, the analysis of MHT-associated breast cancer risk by receptor subtype was then restricted to the subset of women classified as postmenopausal.

1. **Receptor status data extraction, linkage to registry records and the CLEAR questionnaire dataset**

In NSW, for incident breast cancers, a tumor sample obtained through core biopsy (if it was performed for diagnostic purposes) and tumor tissue excised during surgical treatment are usually assessed by pathology laboratories as per the Royal College of Pathologists of Australasia (**RCPA**) guidelines.^2^ This assessment includes the detection of ER, PR, and HER2 protein by ImmunoHistoChemistry (IHC) or HER2 gene amplification by Fluorescent In Situ Hybridization (**FISH**). In NSW, pathology laboratories that perform these tests are required to send a copy of the pathology report to the NSWCR. These pathology reports are scanned and stored in the ‘Workflow’ data repository of the registry.

Receptor status data for the current analysis were obtained through manual data extraction from scanned reports in this repository. In this system, all pathology reports belonging to an individual are stored together; therefore for each woman, reports from fine needle aspiration and/or core biopsy and/or excision biopsy were available. For most cases, there was concordance between the reports. When there was a disagreement, information from the excision biopsy was considered to be the most accurate when designating ER, PR, and HER2 status. The extracted data were then checked by Cancer Institute NSW (CINSW) staff (the data custodians) and de-identified data were made available for linkage with the registry records and CLEAR dataset.

The record linkage process was facilitated by the Centre for Health Record Linkage (CHeReL), which performs record linkage in NSW.^3^ In brief, the process was as follows: Cancer Council NSW (Custodians of CLEAR data) provided CHeReL with an encrypted source record number and identifying details for each breast cancer case in the dataset. CHeReL assigned a Project-specific Person Number (PPN) and performed probabilistic matching of the identifying details to match records from the CLEAR study and the registry records that belonged to the same woman. The PPNs were also used to select the corresponding pathology reports in the NSWCR workflow for data extraction on site at CINSW. We combined the line data for the same woman from the different datasets (in this case, the extracted receptor data, registry data and CLEAR data) using the PPN, which was the person identifier common to all three datasets.

1. **Assessing completeness and representativeness of receptor status information in the NSWCR pathology data repository**

The NSWCR does not routinely enter the tumor receptor information into the registry dataset and so an estimate of the completeness of the receptor information for the breast cancer cases recorded in the registry was not available. Therefore, there was uncertainty about how many of the breast cancer cases participating in the CLEAR study had ER, PR, and HER2 receptor information in the pathology records collection. As a first step towards performing the analysis on MHT use and breast cancer risk by tumor receptor status, we estimated the proportion of CLEAR breast cancer cases in the registry who had information on the ER, PR and HER2 status of their cancers against the total number of CLEAR cases who had a confirmed breast cancer diagnosis in the registry.

The proportions of ER+/PR+ (double receptor-positive), ER+/PR- or ER-/PR+ (single receptor-positive), and ER-/PR- (double receptor-negative) breast cancers were estimated for the subset of CLEAR cases who had receptor status information. Similarly, the proportions of the four subtypes based on ER, PR and HER2 status, i.e. ER+/PR+/HER2- , ER+/PR+/ HER2+ ER-/ PR-/HER2+ and ER-/PR-/ HER2- were also estimated for this dataset. To assess representativeness, these proportions for women in the CLEAR study were compared with those from other reports in the literature, especially those from previous Australian reports.

The population-level proportional distribution of the breast cancer subtypes by receptor status was not available from the registry. However, the proportions by degree of spread were available and used as a surrogate. To ascertain the representativeness of the CLEAR breast cancer cases, we compared the proportions with localised, regional and metastatic breast cancer with the corresponding proportions in the NSW population data for the five-year period 2008-2012.

**S1 Table A - Completeness of receptor information and receptor status for 676 CLEAR breast cancer cases with an ICD10 code of ‘C50’ in the registry**

|  | Proportion complete (%)* | No pathology record | Positive (%) | Negative (%) | Missing /unknown/ awaited | Equivocal |
| --- | --- | --- | --- | --- | --- | --- |
| ER status | 96% | 7 (1%) | 529 (79%) | 119 (18%) | 21 (3%) | 0 (0%) |
| PR status | 96% | 7 (1%) | 464 (69%) | 184 (28%) | 21 (3%) | 0 (0%) |
| HER2 status | 85% | 7 (1%) | 90 (13%) | 462 (69%) | 97 (14%) | 20 (3%) |

* % of the 676 CLEAR breast cancer cases with an ICD10 code of ‘C50’ in the registry

**S1 Table B - Proportional distribution of breast cancer receptor subtypes in cases with known ER, PR, and HER2 status**

| By ER and PR expression only | | | | | |
| --- | --- | --- | --- | --- | --- |
|  | ER+PR+ | ER+/PR- | ER-/PR+ | ER-/PR- | Total |
| All cases | 456 (70%) | 75 (12%) | 10 (1.5%) | 109 (17%) | 650* (100%) |
| Postmenopausal | 277 (66%) | 66 (16%) | 6 (1.4%) | 70 (17%) | 419 (100%) |
| By joint expression of ER, PR, and HER2 | | | | | |
|  | (ER+/PR+ or ER+/PR- or ER-/PR+)  and HER2-  (approximated surrogate for luminal(HER2-)) | (ER+/PR+ or ER+/PR- or ER-/PR+)  and HER2+  (approximated surrogate for luminal(HER2+)) | ER-/PR-  and  HER2+  (surrogate for HER2 type) | ER-/PR- and  HER2-  (Triple negative/ surrogate for basal-like) | Total |
| All cases | 396 (72%) | 55 (10%) | 35 (6%) | 65 (12%) | 551^#^ (100%) |
| Postmenopausal | 245 (70%) | 38 (11%) | 25 (7%) | 40 (11%) | 348 (100%) |

* 21 cases had missing ER and PR information; percentages shown are within the subset of cases with complete information. ^#^ 20 cases had equivocal results, 97 cases had unknown or missing value for HER2 status.

**S1 Table C - Proportional distribution of breast cancers by degree of spread in the overall CLEAR study dataset and in the NSW Cancer Registry**

| Degree of spread | CLEAR study dataset | NSW Cancer Registry |
| --- | --- | --- |
| Local | 55% | 50% |
| Region | 40% | 38% |
| Metastatic | 3% | 7% |
| Unknown | 2% | 5% |

*Data are available from the Cancer Council New South Wales Institutional Data Access/ Ethics Committee for researchers who meet the criteria for access to confidential data. Data are from the NSW CLEAR study whose authors may be contacted at Cancer Council New South Wales through the Chief Investigator Prof Karen Canfell at* [*karen.canfell@nswcc.org.au*](mailto:karen.canfell@nswcc.org.au) *or via the institutional email at CancerResearch@nswcc.org.au*”

1. **References**
2. Beral V and Million Women Study Collaborators. Breast cancer and hormone-replacement therapy in the Million Women Study. Lancet 2003: 362 (9382); 419-427
3. The Royal College of Pathologists of Australasia. Invasive breast cancer structured reporting protocol (2nd edition, 2012). Accessed at https://[www.rcpa.edu.au/getattachment/7b70b3e5-5dca-403f-893e-638815f487b1/Protocol-invasive-breast-cancer.aspx](http://www.rcpa.edu.au/getattachment/7b70b3e5-5dca-403f-893e-638815f487b1/Protocol-invasive-breast-cancer.aspx) on 23 May 2018.
4. The Centre for Health Record Linkage. How record linkage works. Accessed at <http://www.cherel.org.au/how-record-linkage-works> on 23 May 2018.
